# Tumour‐proximal deep serum proteomics identifies biomarkers associated with ovarian cancer

**DOI:** 10.1002/1878-0261.70351

**Published:** 2026-10-17

**Authors:** Darren Lau, Daniel Pincher, Clifford Young, Nicholas Chappell, Noor A. Lokman, Parul Mittal, Leigh Donnellan, Martin K. Oehler, Doug A. Brooks, Peter Hoffmann

**Affiliations:** ^1^ School of Pharmacy and Biomedical Science, College of Health Adelaide University Adelaide Australia; ^2^ School of Medicine, College of Health Adelaide University Adelaide Australia; ^3^ Robinson Research Institute, College of Health Adelaide University Adelaide Australia; ^4^ Department of Gynaecological Oncology Royal Adelaide Hospital Adelaide Australia; ^5^ Discipline of Histopathology, School of Medicine Trinity Translational Medicine Institute, Trinity College Dublin Dublin Ireland

**Keywords:** malignancy, nanoparticle enrichment, ovarian and peripheral vein serum, ovarian cancer, protein biomarkers, proteomics

## Abstract

Ovarian cancer diagnosis is often delayed due to poorly defined clinical symptoms, underscoring the need for novel biomarkers that enable earlier detection. Protein biomarker discovery in blood samples is analytically challenging using mass spectrometry, as high‐abundance proteins can obscure detection of low‐abundance biomarkers. Ovarian vein (ovarian) and median cubital vein (peripheral) serum were collected from healthy women or patients with benign or malignant (majority stage I) serous ovarian tumours. Proteograph XT assay was employed as a protein enrichment methodology prior to LC–MS/MS analyses to obtain deeper proteome coverage in these samples. From an initial cohort of 53 samples, we identified 6603 proteins across all samples, including 3467 proteins uniquely identified in ovarian serum. Following exclusion of red blood cell–contaminated samples, differential abundance analysis identified 86 and 101 malignancy‐associated protein candidates in ovarian and peripheral samples, respectively. Notably, ANK1, CAND1 and GMPR2 were differentially abundant across both sample types. Using logistic regression with repeated cross‐validation and permutation testing, ANK1 demonstrated strong discriminatory performance [area under the curve (AUC) = 0.97] when combined with clinical CA125 measurements. These findings highlight the importance of ovarian vein sampling for enhanced access to tumour‐associated proteins and support further validation of these candidate biomarkers for early ovarian cancer detection.

## Graphical abstract

New biomarkers are needed for early ovarian cancer detection. Ovarian (tumour‐proximal) and peripheral vein serum from healthy women and patients with benign or malignant tumours underwent nanoparticle‐enriched deep proteomics. Ovarian serum yielded more protein identifications and revealed tumour‐reflective signatures. ANK1, CAND1 and GMPR2 were differentially abundant in both sample types, and ANK1 combined with CA125 achieved an AUC of 0.97.

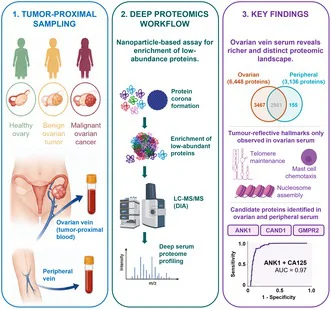

**Abbreviations**
ACTB: actin B
ANK1: ankyrin‐1
AUC: area under the curve
BDNF: brain‐derived neurotrophic factor
CA125: cancer antigen 125
CAND1: cullin‐associated and neddylation‐dissociated protein 1
CCD80: coiled‐coil domain containing 80
CCL18: C‐C motif chemokine ligand 18
CID: collision‐induced dissociation
CPTAC: Clinical Proteomic Tumour Analysis Consortium
CV: coefficient of variation
DIA: data‐independent acquisition
ELISA: enzyme‐linked immunosorbent assay
EPB41: erythrocyte membrane protein band 4.1
FDR: false discovery rate
FN3K: fructosamine‐3‐kinase
GMPR2: guanosine monophosphate reductase 2
HE4: human epididymis protein 4
HEM3: hydroxymethylbilane synthase
HREC: Human Research Ethics Committee
ITB3: integrin beta‐3
KPRB: phosphoribosyl pyrophosphate synthetase associated protein 2
LC–MS/MS: liquid chromatography tandem mass spectrometry
MUC16: mucin‐16
npA: nanoparticle A
npB: nanoparticle B
PASEF: parallel accumulation serial fragmentation
PCA: principal component analysis
RAC2: Ras‐related C3 botulinum toxin substrate 2
RAH: Royal Adelaide Hospital
ROC: receiver operating characteristic
TIMS: trapped ion mobility spectrometry
WFDC2: WAP four‐disulfide core domain protein 2

## 1 Introduction

Ovarian cancer is the eighth most common cancer among women worldwide, accounting for more than 324 000 new cases and over 206 000 deaths annually [1]. Approximately 90% of ovarian cancers have an epithelial cell origin, of which ~70% are histotyped as high‐grade serous ovarian carcinomas [2]. Ovarian cancer is considered early stage when the disease is confined to the ovaries or nearby pelvic structures, including: Stage I, where the tumour is limited to one or both of the ovaries (or fallopian tubes); and Stage II, where the tumour has extensions to the uterus or other pelvic organs (e.g. fallopian tubes, bladder or rectum), but no spread outside of the pelvis. For the treatment of early‐stage ovarian cancers, the combination of surgery and adjuvant platinum‐based chemotherapy results in a 5‐year survival rate of between 70 and 95%, but this survival rate falls below 40% for patients with advanced stage disease [3]. Advanced Stages III and IV are characterized by tumour metastasis, occurring inside and outside the extra pelvic peritoneum, respectively [4]. Approximately 75% of patients with ovarian cancer are diagnosed at an advanced stage and <20% of these patients survive longer than 10 years [5]. Early detection of ovarian cancer is therefore important for improving treatment outcomes and survival rates, but many factors currently impede this critical objective.

The early diagnosis of ovarian cancer is currently challenging because the disease typically presents with vague, nonspecific symptoms and there is a lack of reliable technology for accurate detection. Approximately 95% of patients experience nonspecific symptoms before diagnosis, which include abdominal bloating, gastrointestinal disturbances and urinary urgency [6]. These symptoms are commonly observed with other illnesses, such as irritable bowel syndrome, and therefore are not specific for ovarian cancer diagnosis [7]. The detection of ovarian cancer by the quantitation of mucin 16 (MUC16), commonly known as cancer antigen 125 (CA125) protein, in peripheral blood samples lacks both sensitivity due to low expression in early‐stage disease and specificity as it is elevated in many benign conditions (endometriosis, menstruation, pregnancy and pelvic inflammatory disease) [8]. In comparison, transvaginal ultrasound can have higher sensitivity for detecting ovarian masses and structural abnormalities, but lacks specificity because of other benign conditions (cysts, endometriomas and fibroids), which may result in false‐positive findings [9]. Importantly, these approaches for ovarian cancer detection were not found to significantly reduce ovarian cancer mortality in asymptomatic women [1, 10].

Many genetic abnormalities are involved in the pathogenesis of ovarian cancer, which include common mutations that activate proto‐oncogenes (*KRAS* and *MYC*) or inactivate tumour suppressor genes (*BRCA1, BRCA2* and *TP53*) [11]. The highly complex molecular genetics landscape together with marked genomic instability likely alters the function of many proteins in ovarian cancer and that any effective detection system will rely on a greater understanding of the proteins and cellular pathways involved. Global analysis by a proteomics approach is ideally suited to investigate the tumour complexity in ovarian cancer samples [12]. The normal and ovarian cancer blood serum proteome is primarily composed of albumin/albumin‐like proteins, apolipoproteins, acute phase proteins and coagulation proteins, as well as numerous tissue leakage and signalling proteins [13]. All organs contribute to the protein component of blood, making tumour‐specific markers potentially difficult to identify, but some proteins can still exhibit quantitative changes in a disease‐specific manner [14]. Blood/serum samples are a minimally invasive sample type that potentially contains specific protein biomarkers originating from the tumour. However, quantitative protein analysis (and therefore protein biomarker discovery) of human blood samples is an analytically challenging task, largely due to the presence of the aforementioned high‐abundance proteins. It is generally accepted that serum protein abundances span at least 10 orders of magnitude and the 22 most abundant proteins constitute approximately 99% of the total protein content [15]. Additionally, the dynamic range of the lowest abundant serum proteins is beyond the detection capabilities of current mass spectrometry instrumentation without a form of enrichment [16]. Sample collection and storage must also be conducted carefully to minimize the presence of contaminating proteins from red blood cell lysis, platelets and partial coagulation [17].

In order to achieve deeper proteomic coverage, numerous strategies were utilized to deplete the high‐abundance proteins, including immunodepletion [18, 19, 20], precipitation [21, 22] or multidimensional fractionation [23], to extensively separate serum proteins. Several affinity‐based technologies have recently emerged that provide previously unprecedented human serum proteomic coverage and extensive opportunities for biomarker discovery, which involve the use of SomaScan aptamer‐based microarrays [24] or Olink proximity extension assays [25]. The latter technology previously permitted the discovery of ovarian protein biomarkers in the plasma of women with malignant tumours compared with benign lesions [26, 27, 28, 29, 30]. Despite their high sensitivity for low‐abundance circulating proteins, these technologies are generally limited to predefined targets, thus restricting detection to what the panel was designed to capture. Another promising methodology involves the enrichment of serum proteins by protein corona formation on magnetic nanoparticles prior to measurement by mass spectrometry analysis [31]. Recent comparisons involving many of the aforementioned techniques have largely revealed complementary results, despite all of them achieving deeper proteomic coverage [32, 33]. For example, Olink and nanoparticle enrichment via Seer Proteograph XT assay identified almost 6000 protein groups in plasma samples, while Somascan detected up to almost 10 000 protein groups [33]. The Proteograph assay facilitates protein enrichment via the surface chemistry on two physicochemically distinct nanoparticle fractions to promote protein corona formation upon serum sample contact, with highly abundant proteins slowly replaced by lower abundance proteins with higher affinity [34]. Due to the distinct physicochemical surface properties of the two nanoparticle fractions A (npA) and B (npB), they are expected to enrich nonidentical subsets of the serum proteome. As a result, differences in protein identification and abundance between the two nanoparticle assays are expected, and consistent trends across both datasets may provide greater confidence in their reproducibility.

In this study, we conducted a deep proteomic analysis by inducing protein corona formation from commercially available nanoparticles on serum samples collected from the ovarian and median cubital veins of healthy women and patients with benign or malignant ovarian cancer. Due to its close vicinity to the ovary, the protein profiles of ovarian vein samples may reveal potential novel protein biomarkers that were enriched (due to close proximity) and originated from the tumour or tumour microenvironment [35]. Cubital (or peripheral) serum was analysed in parallel to enable the discovery of tumour biology‐relevant, peripheral blood‐based biomarkers for ovarian cancer detection, with both datasets interrogated for common candidates. Our analysis delineated distinct proteome profiles between ovarian and peripheral vein samples. However, after exclusion of haemolysed blood samples, we identified candidate proteins and biological pathways that were associated with ovarian cancer malignancy in both sample types. Three protein biomarkers were consistently identified across ovarian and peripheral serum and demonstrated good discriminatory performance for malignancy. Collectively, this work provides a foundation for future validation studies aimed at developing clinically relevant ovarian cancer diagnosis biomarkers to improve early detection.

## 2 Materials and methods

### 2.1 Ethics approval and serum sample collection

This study involved human participants and was conducted with written informed consent and approval by the Royal Adelaide Hospital (RAH; Adelaide, Australia) Human Research Ethics Committee (HREC) under RAH protocol or HREC reference numbers 080102, 140101 and HREC/18/CALHN/811. The study methodologies conformed to the standards set by the Declaration of Helsinki. A total of 53 serum samples were collected between 2012 and 2020 for this study at the RAH following written informed consent from patients undergoing surgery for gynaecological disease. Blood samples were collected from the ovarian veins of 1 healthy, 11 benign and 9 malignant (Stages Ia to III) patients [35], with corresponding peripheral blood samples collected from the median cubital vein. In addition, 1 ovarian and 10 peripheral blood samples were collected from unrelated healthy volunteers undergoing surgery for other gynaecological diseases. Serum CA125 concentrations were determined using the Siemens ADVIA Centaur XP automated analyser at SA Pathology, Adelaide, Australia, in accordance with the manufacturer's instructions and established laboratory quality control procedures (Table S1). Additional details including sampling time and associated patient status can be found in Table S1. All blood samples were collected into clotting tubes (Greiner Bio‐one), and serum was prepared by centrifugation at 3000 rpm for 15 min at room temperature. The resulting supernatant was aliquoted and stored at −80°C until analysis.

### 2.2 Proteograph XT assay for serum protein enrichment

Serum protein enrichment was performed using reagents from the Proteograph XT Assay Kit in conjunction with a SP100 Automation Instrument (SEER) as per the manufacturer's instructions. Briefly, serum samples were thawed and centrifuged at 5000 ***g*** for 2 min. Approximately 240 μL of serum was dispensed into a fresh sample tube before centrifugation at 5000 ***g*** for 1 min. After being loaded into the SP100 Automation Instrument, neat serum samples were incubated with two types of proprietary paramagnetic nanoparticles (npA and npB) for 1 h to facilitate stable protein corona formation. Following magnetic collection, the nanoparticles were washed and the protein coronas subjected to denaturation, reduction and alkylation prior to proteolytic digestion. The resulting peptides were desalted using solid phase extraction, and the eluates were dried at 50 °C in a vacuum centrifuge before storage at −20 °C. After peptide quantification by fluorescence using the Pierce BCA Modified Peptide Kit (#23290; Thermo Fisher Scientific, MA, USA), samples were resuspended in 0.1% formic acid prior to LC–MS/MS analysis.

### 2.3 LC–MS/MS

LC–MS/MS analysis was conducted on nanoparticle‐enriched samples (npA and npB) using separate injections. The data‐independent acquisition (DIA) method was performed on the timsTOF Flex instrument (dia‐PASEF, DIA parallel accumulation serial fragmentation; Bruker Daltonik, Bremen, Germany) with a nanoelectrospray Captive Spray ion source (Bruker Daltonik) and a 20 μm Classic Emitter (Bruker Daltonik). The system was coupled online to an UltiMate 3000 nanoLC instrument (Thermo Fisher Scientific). The instrument was operated using Bruker Compass 4.1 for the timsTOF series (Control Version 6.2, Build 1.2, 64‐bit) and Compass HyStar 6.3 (Version 6.3.1.8), with Thermo Scientific™ Dionex™ Chromeleon™ 7 Chromatography Data System (Version 7.3). Prior to analysis, the instrument was calibrated using Agilent ESI‐L Low Concentration tuning mix (Agilent Technologies, CA, USA), with the ion mobility (IM) dimension calibrated linearly using three reference ions (*m/z*, 1/K0: 622.0289, 0.9848 Vs·cm^−2^; 922.0097, 1.1895 Vs·cm^−2^; and 1221.9906, 1.3820 Vs·cm^−2^).

For LC separation, 200 ng peptides from each nanoparticle‐enriched sample were injected onto a PremierLCMS 150 μm ID × 150 mm (1.5 μm, 100 Å Reprosil Saphir C18) reversed‐phase column. The flow rate was maintained at 1 μL·min^−1^, with the column compartment heated to 40 °C. Mobile phase A was 0.1% formic acid in water, and mobile phase B was 0.1% formic acid in 80% acetonitrile. The gradient began with a linear increase of mobile phase B from 3.8% to 25% over 45 min, followed by an increase to 87.5% in 3 min, which was held for 5 min, leading to a total run time of 70 min.

The dia‐PASEF method, as described by Meier *et al*. [36], was employed. Briefly, the MS scan range was 100–1700 *m/z*, with an IM window of 1/K0 0.6–1.6, 24 MSMS window, a ramp time of 100 ms and a duty cycle of 100%. The accumulation time was 2 ms, capillary voltage was set to 1500 V, dry gas flow was 3.0 L·min^−1^, and dry temperature was 180 °C. Additional settings included a deflection 1 delta of 70.0 V, funnel 1 RF of 300.0 Vpp, funnel 2 RF of 200.0 Vpp and CID energy of 0.0 eV. TIMS‐specific parameters included Δt1 of −20.0 V, Δt2 of −160 V, Δt3 of 110.0 V, Δt4 of 110.0 V, Δt5 of 0.0 V, Δt6 of 55.0 V, Funnel 1 RF of 450.0 Vpp and a collision cell in voltage of 300.0 V.

### 2.4 Data analysis and visualization

Raw data were processed using the Spectrometer software (v19; Biognosys, Zurich, Switzerland), in library‐free mode (directDIA™) with default settings. For peptide identifications, the data were searched against the *Homo sapiens* FASTA file containing 20 596 protein entries (UniProt release 2023_05). Trypsin/P was selected as the digestion enzyme, and a maximum of two missed cleavages were permitted. Variable modifications included protein N‐terminal acetylation and methionine oxidation, while cysteine carbamidomethylation was a fixed modification. Filtering criteria included a 1% false discovery rate (FDR) at the peptide‐spectrum match (PSM) and peptide and protein group levels. Coefficients of variation in percentage (%CV) were calculated for each protein group across biological replicate measurements (*n* stated in each figure) as the standard deviation divided by the mean, multiplied by 100%. The median %CV across all protein groups was then calculated to provide an overall measure of variability. Gene Ontology analysis was conducted using the Database for Annotation, Visualization and Integrated Discovery (DAVID, https://davidbioinformatics.nih.gov/) tool, with significance determined using Benjamini–Hochberg adjusted *P*‐values. For the investigation of biological processes associated with malignancy, Gene Ontology analyses were performed using the candidate proteins identified across both nanoparticle datasets. Candidate proteins showing increased and decreased abundance were analysed separately, with the union of proteins detected across the npA and npB datasets used as the background protein set. Red blood cell contamination of serum samples was assessed using the Baize software (https://www.guomics.com/software/Baize) [37]. Unpaired Student *t*‐tests were used for all pairwise comparisons, with differentially abundant proteins considered using a log_2_ fold change cut‐off of 0.58 and FDR‐adjusted *P*‐value (or *q*‐value) less than 0.05. Candidate biomarkers were further filtered using an additional criterion requiring detection in at least 80% of samples within the relevant dataset. Area under the curve (AUC) values for single candidate biomarkers were determined using univariate receiver operating characteristic (ROC) curve analysis. Selected protein candidates were subsequently evaluated individually and in combination with clinical CA125 data using logistic regression models implemented in the Tester module of MetaboAnalyst 6.0. Data were log_2_‐transformed and predictive performance was evaluated using the default Monte Carlo cross‐validation (100 runs) fold settings implemented in MetaboAnalyst 6.0, with each run randomly partitioning the data into 2/3 training and 1/3 test sets. Predictive accuracy was reported as the average across repeated cross‐validation runs. Statistical significance was assessed using permutation testing (1000 permutations). ROC curves were plotted using Excel. Line and bar graphs, violin plots and boxplots were plotted using GraphPad Prism (version 10.0.2).

## 3 Results

### 3.1 Proteomic analysis of serum samples derived from median cubital and ovarian veins

The composition of the study cohort is illustrated in Fig. 1A. Serum samples were processed using the Proteograph XT assay to enhance the detection of low‐abundance proteins. Each sample was analysed using two nanoparticles (referred to as nanoparticle A, npA and nanoparticle B, npB), resulting in a total of 106 samples analysed by LC–MS/MS.

**Fig. 1:**
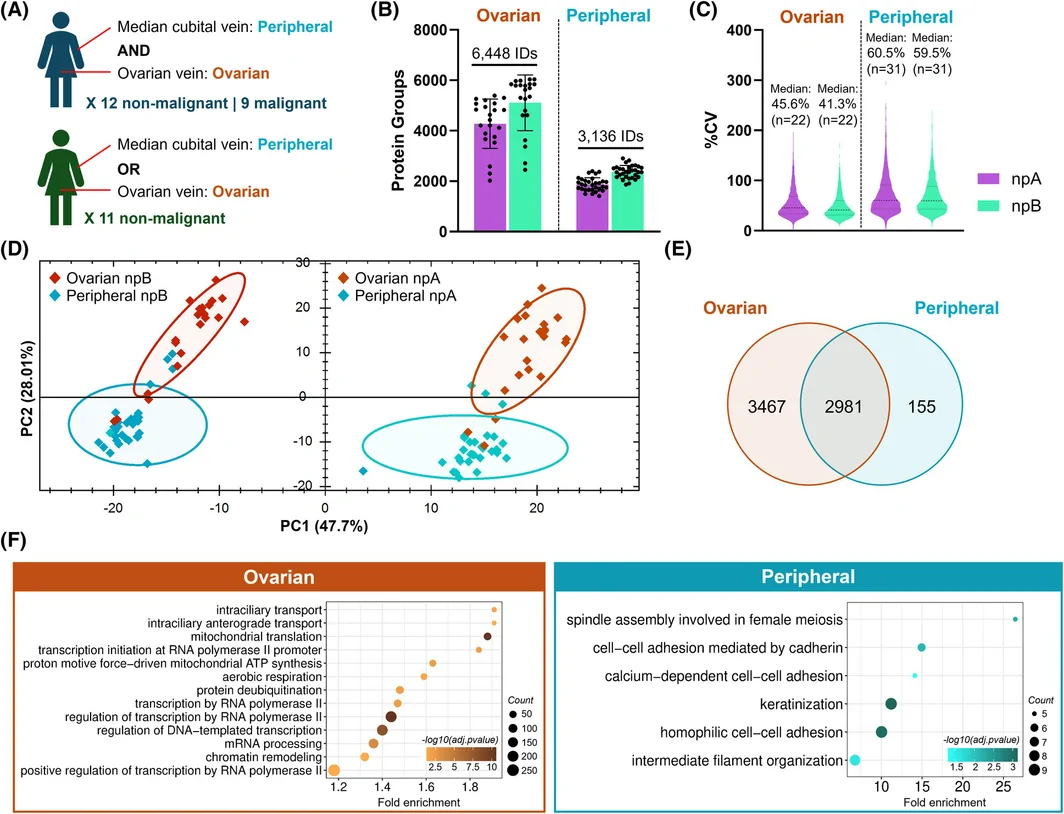
Proteomic analysis of serum samples derived from ovarian and median cubital veins. (A) Composition of initial study cohort (see the Methods section). (B) Number of protein groups identified from ovarian (*n* = 22) and peripheral (*n* = 31) samples by Proteograph XT assay nanoparticles A (npA) and B (npB). Results presented as mean ± SD. (C) Coefficient of variations by percentage (%CV) for protein groups identified in ovarian and peripheral samples by Proteograph XT assay npA and npB. (D) Principal component analysis (PCA) of peripheral and ovarian sample proteome profiles by npA (*positive PC1 axis*) and npB (*negative PC1 axis*). (E) Venn diagram showing the number of unique proteins identified in ovarian and peripheral samples. (F) Gene Ontology analysis of biological pathways enriched using protein groups unique to sample types against a background of 6603 proteins identified in ovarian and peripheral serum using npA and npB, with significance determined using Benjamini–Hochberg adjusted *P*‐values.

Ovarian serum samples yielded a substantially higher number of protein group identifications (>6400) as compared with peripheral samples (>3100) (Fig. 1B) when processed with matching between runs. Consistent with this, variability differed between sample types, with median %CV of approximately 40% for ovarian samples and 60% for peripheral samples (Fig. 1C). Principal component analysis (PCA) highlighted a distinct proteome profile between npA and npB datasets. Within each nanoparticle dataset, peripheral and ovarian samples clustered separately, indicating tissue‐specific differences in proteomic profiles (Fig. 1D).

A total of 6603 unique proteins were identified across both sample types, with 3467 and 155 protein groups found to be unique to ovarian and peripheral samples, respectively (Fig. 1E). We compared these 6603 proteins with the predefined protein lists for the Olink Explore HT (https://olink.com/products/olink‐explore‐ht) and SomaScan 11K assays [38]. Consistent with previous studies [32, 33], only limited overlap was observed (Fig. S1), highlighting the complementary nature of these analytical platforms.

Gene Ontology analysis further highlighted functional differences between the sample types. Proteins uniquely identified in ovarian samples (3467 protein groups) were enriched for biological processes related to mRNA processing, ATP synthesis or respiration, and exocytosis. In contrast, proteins unique to peripheral samples (155 protein groups) were enriched with pathways related to epithelial cell development and differentiation, spindle assembly and cell adhesion (Fig. 1F).

We further assessed the number of protein groups detected with each assay in each ovarian (Fig. S2A) and peripheral (Fig. S2B) serum sample. In general, npB consistently yielded a greater number of protein group identifications than npA in both sample types. When npA and npB datasets were processed independently (without match‐between‐runs), ovarian serum samples yielded approximately 4600 and 5808 protein groups from npA and npB, respectively, with 3779 protein groups detected in common between the two nanoparticle types (Fig. S2C). Similarly, in peripheral serum samples, npA and npB identified 2183 and 3054 protein groups, respectively, with 1767 overlapping protein groups (Fig. S2D). Among commonly detected proteins, abundance measurements showed weak correlations between npA and npB datasets (ovarian: *R* ^2^ = 0.4055, *P* < 0.001; peripheral: *R* ^2^ = 0.4325, *P* < 0.001) (Fig. S2E–F). Overall, these findings highlight that both nanoparticle types should be considered as independent assays for downstream data processing and analysis.

Given the considerable variations in protein group identifications and noticeable presence of red blood cell haemolysis, we further investigated potential red blood cell contamination that may introduce variability into proteome profiles of serum samples and thus compromise quantification. Using a recently developed open‐access software Baize for contamination assessment [37], we identified two peripheral and seven ovarian samples to be affected by red blood cell contamination (Fig. 2A). Interestingly, samples flagged as contaminated by Baize were consistent with visual observations, as these samples typically exhibited a darker red coloration. However, reliance on visual inspection alone may introduce bias, as some samples displayed an intermediate reddish orange coloration that could be perceived as contaminated (Fig. S3). After exclusion of contaminated samples, this resulted in 5558 and 3003 protein group identifications across ovarian and peripheral samples, respectively (Fig. 2B; Tables S3, S4). The median %CV ranged from approximately 40 to 50% across both sample types (Fig. 2C), which is an improvement in comparison with the original dataset.

**Fig. 2:**
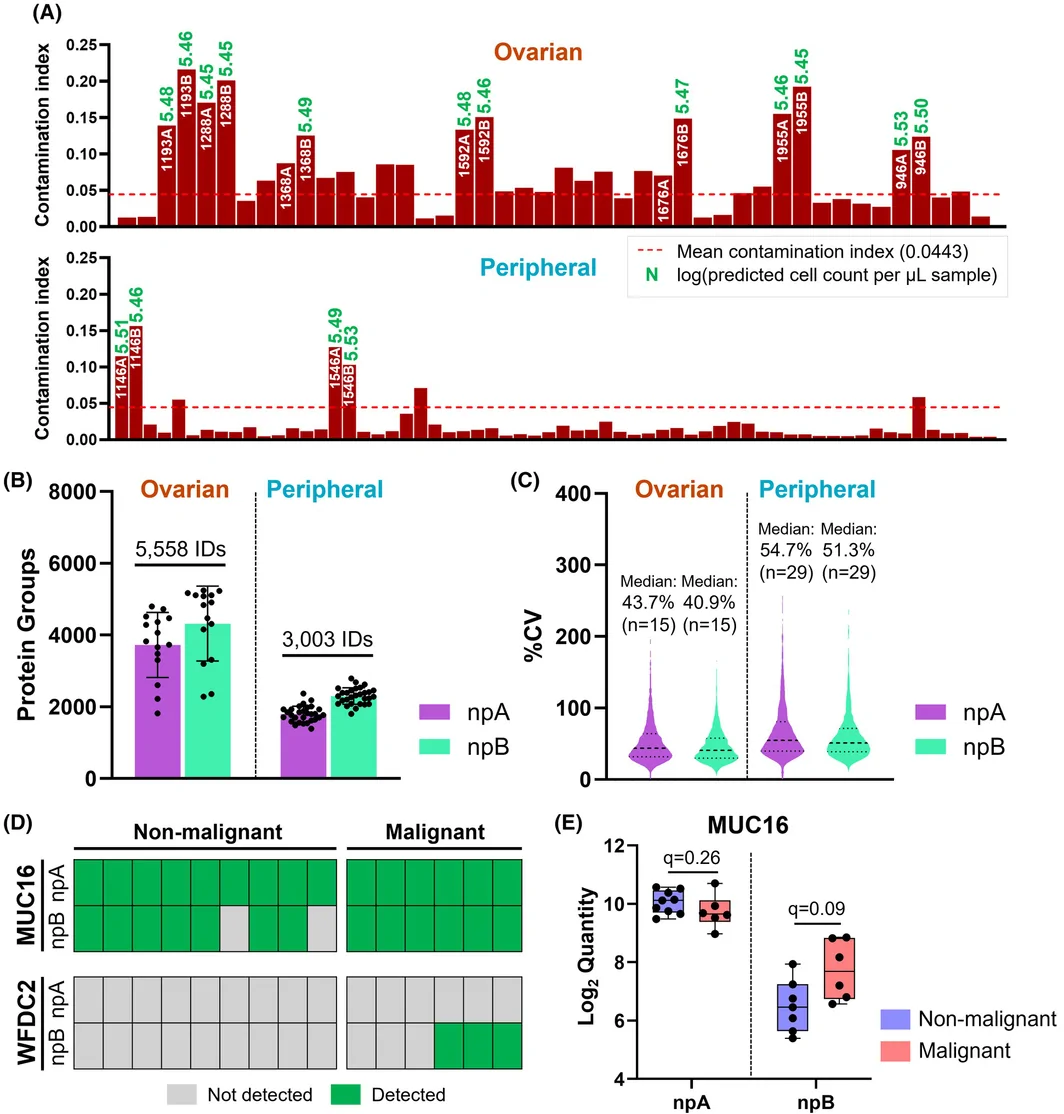
Subsequent analysis post quality control. (A) Evaluation of blood contamination using the Baize software. Bar graphs displaying the contamination index for each sample. Contamination indexes were based on the quantity of protein contaminant biomarkers compared with total protein. The predicted cell count per μL of sample is stated in green above samples that were flagged for contamination. Sample ID and nanoparticle counterparts for contaminated samples (excluded from study for further analysis) are labelled in white. Red dotted line represents mean contamination index of 0.0443. (B) Number and (C) coefficient of variation in percentage (%CV) of protein group identifications from ovarian (*n* = 15) and peripheral samples (*n* = 29) by Proteograph XT assay nanoparticles A (npA) and B (npB) after exclusion of blood‐contaminated samples. Error bars represent mean ± SD. (D) Detection of MUC16 and WFDC2 proteins in nonmalignant (*n* = 9) and malignant (*n* = 6) ovarian serum samples without blood contamination. Each column represents one patient. (E) Boxplot comparing the log_2_‐transformed quantities of MUC16 between nonmalignant and malignant ovarian serum samples identified using npA and npB. Significance was assessed using false discovery rate (FDR)‐controlled unpaired Student's *t*‐tests, with a threshold of *q* < 0.05.

We also assessed the detection of currently established clinical biomarkers for ovarian cancer, such as mucin‐16 (MUC16) and WAP four‐disulfide core domain protein 2 (WFDC2), commonly associated with CA125 and human epididymis protein 4 (HE4), respectively. Both MUC16 and WFDC2 were detected exclusively in ovarian vein serum samples (Fig. 2D; Tables S3, S4). However, WFDC2 showed more limited detection than MUC16, being identified in only 20% of ovarian samples in the npB dataset, all of which were from malignant cases (Fig. 2D). In contrast, MUC16 was present in both nanoparticle datasets, with higher abundance recorded in malignant cases using npB (Fig. 2E). Given that CA125 is an epitope associated with tandem‐repeat regions of MUC16, we examined the relationship between available clinical CA125 measurements from peripheral serum and abundances of MUC16‐derived peptides measured in matched ovarian serum samples [39, 40]. Five distinct peptides associated with tandem‐repeat regions of MUC16 were identified in our proteomic analysis, with peptide PDPTGPGLDR dominating the npA dataset (Table S2). Among these, LTLLRPEK, NTSVGPLYSGCR, DGAATGVDAICTHR and ELGPYTLDR are present in at least 5 of 19 tandem repeats of MUC16 [39, 40]. In the npB dataset, these peptides showed strong positive Spearman correlations with matched clinical peripheral CA125 measurements, except for LTLLRPEK (Fig. S4). This suggests that, among the two nanoparticle datasets, MUC16 measurements obtained using npB may be more closely reflecting biological variations associated with clinical CA125 levels.

### 3.2 Differentially abundant proteins between nonmalignant and malignant cases in ovarian vein serum samples

Following cohort validation, we first investigated the differences between malignant and nonmalignant ovarian vein samples. PCA showed considerable overlap between nonmalignant and malignant groups for both npA and npB datasets (Fig. 3A). In the npA dataset, 33 proteins were upregulated in malignant cases, whereas 46 were downregulated when compared with nonmalignant cases (Fig. 3B). Similarly, in the npB dataset, 26 proteins were upregulated in malignant cases, while 44 were downregulated (Fig. 3C). Gene Ontology analysis of proteins upregulated in malignant cases highlighted an enrichment of biological processes related to chromatin organization, megakaryocyte differentiation and nucleosome assembly. In contrast, proteins significantly downregulated were related to RNA and protein localization to Cajal bodies, telomere maintenance, mast cell chemotaxis and actin filament organization (Fig. 3D).

**Fig. 3:**
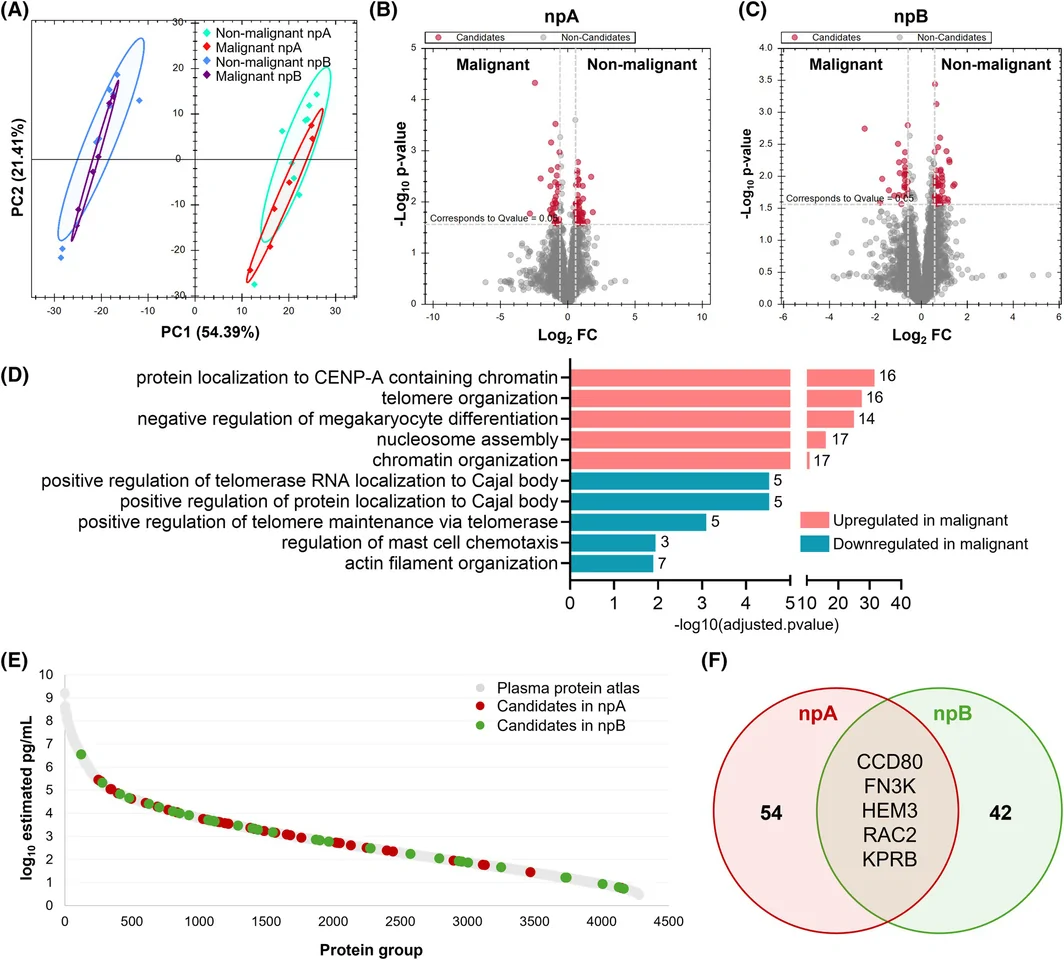
Differentially abundant proteins between nonmalignant and malignant cases in ovarian vein serum samples. (A) Principal component analysis (PCA) plot for proteomes of nonmalignant vs malignant ovarian vein serum samples by nanoparticle types. (B‐C) Volcano plots highlighting malignancy‐associated candidates for npA and npB datasets. Significance was assessed using false discovery rate (FDR)‐controlled unpaired Student's *t*‐tests. (D) Gene Ontology analysis for significantly different abundant proteins between malignant and nonmalignant cases against a background of 5558 proteins listed in Table S3, with significance determined using Benjamini–Hochberg adjusted *P*‐values. The number of proteins associated with each term is indicated on the right. (E) Scatter plot showing log_10_ estimated pg/mL for each potential biomarker identified using npA (*red dots*) and npB (*green dots*), matched against proteins listed in the Human Plasma Protein Atlas (https://www.proteinatlas.org/humanproteome/blood; *grey dots*). (F) Venn diagram represents overlapping of potential protein biomarkers identified across npA and npB datasets.

For biomarker discovery, we applied a more stringent filtering criterion of ≥80% data completeness for each nanoparticle type in which a candidate protein was observed. To provide context for the dynamic range of proteins detected by our workflow, identified proteins were compared with reported circulating protein concentration data from the Human Plasma Proteome Database. This filtering strategy identified 101 unique candidate proteins across both npA and npB datasets, spanning several orders of magnitude in abundance (Fig. 3E; Table S3), highlighting the effectiveness of the Proteograph assay in enriching and identifying low‐abundant protein biomarker candidates. The top 10 malignancy‐associated biomarkers by nanoparticle type, ranked by fold change, are shown in Fig. S5. Notably, only five proteins—coiled‐coil domain containing 80 (CCD80), fructosamine‐3‐kinase (FN3K), hydroxymethylbilane synthase (HEM3), Ras‐related C3 botulinum toxin substrate 2 (RAC2) and phosphoribosyl pyrophosphate synthetase associated protein 2 (KPRB) were consistently differentially abundant across both npA and npB datasets (Fig. 3F; Fig. S6). The minimal overlapping in identified candidates between npA and npB datasets highlight that each nanoparticle type functions as an independent assay. Hence, the consistency of CCD80, FN3K, HEM3, RAC2 and KPRB strengthens their potential as candidate biomarkers.

### 3.3 Differentially abundant proteins between nonmalignant and malignant cases in peripheral serum samples

Consistent with the ovarian vein samples, PCA of peripheral serum proteomes showed considerable overlap and no clear separation between nonmalignant and malignant samples across both nanoparticle datasets (Fig. 4A). In the npA dataset, differential abundance analysis identified a strong imbalance between groups, with 81 protein groups downregulated in malignant cases compared with only four that were upregulated (Fig. 4B). A similar trend was observed for the npB dataset, with 55 and 13 proteins downregulated and upregulated in malignant cases, respectively (Fig. 4C). Proteins upregulated in malignant cases were associated with nuclear localization and negative regulation of apoptotic signalling. On the contrary, proteins downregulated in malignant cases were significantly enriched for pathways linked to protein stability and apoptosis (Fig. 4D). The same stringency (≥80% data completeness) was applied for biomarker discovery, resulting in 86 unique candidate proteins spanning several orders of magnitude in abundance, albeit with fewer candidates detected at the lowest abundance range (Fig. 4E; Table S4). This further supports the capability of the Proteograph assay to capture a broad dynamic range of protein biomarkers. The top 10 malignancy‐associated biomarkers for each nanoparticle type, ranked by fold change, are presented in Fig. S7. Similar to the ovarian vein samples, the Venn diagram shows a minor overlap of four proteins (BDNF, ITB3, EPB41 and ACTB) between the npA and npB protein candidate lists, with consistent abundance trends in both datasets (Fig. 4F; Fig. S8).

**Fig. 4:**
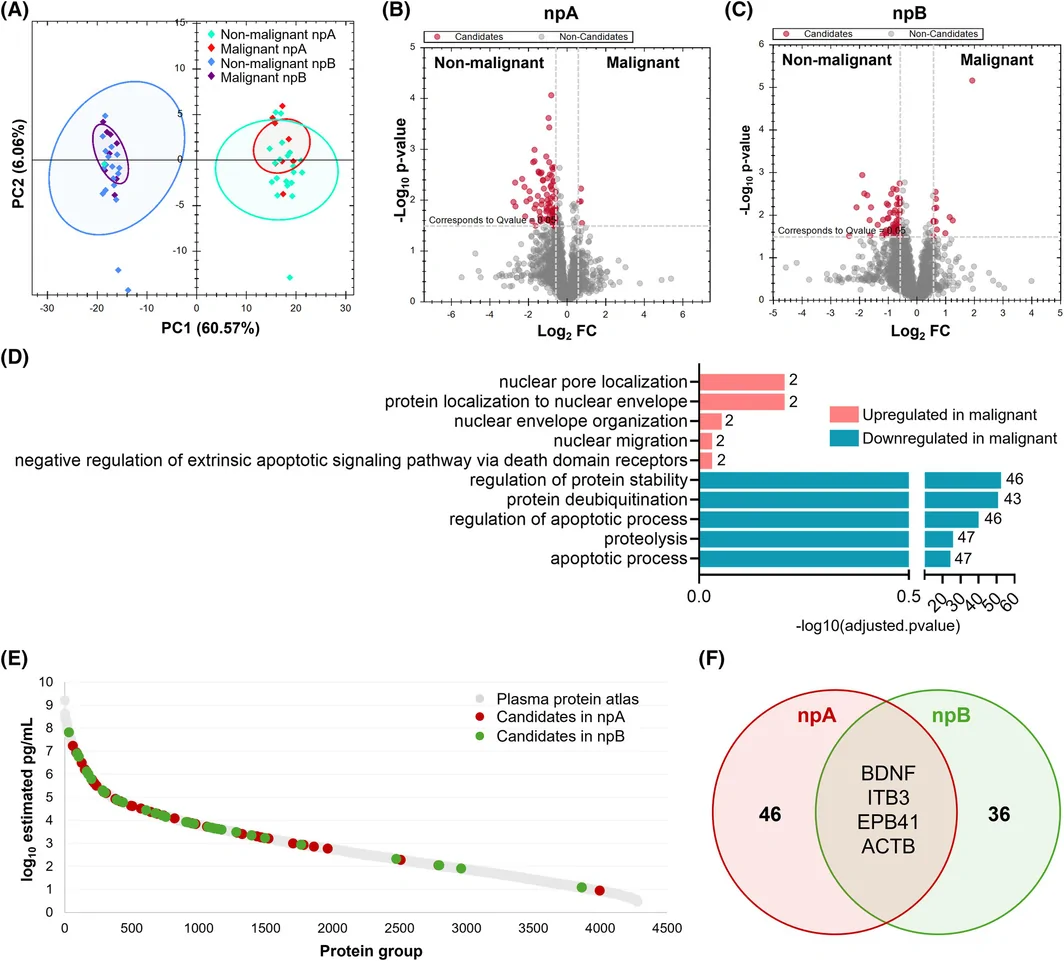
Differentially abundant proteins between nonmalignant and malignant cases in median cubital vein peripheral serum samples. (A) Principal component analysis (PCA) plot for proteomes of nonmalignant vs malignant peripheral serum samples by nanoparticle types. (B, C) Volcano plots highlighting malignancy‐associated candidates for npA and npB datasets. Significance was assessed using false discovery rate (FDR)‐controlled unpaired Student's *t*‐tests. (D) Gene Ontology analysis for proteins showing significant differential abundance between malignant and nonmalignant cases against a background of 3003 proteins listed in Table S4, with significance determined using Benjamini–Hochberg adjusted *P*‐values. The number of proteins associated with each term is indicated on the right. (E) Scatter plot showing log_10_ estimated pg/mL for each potential biomarker identified using npA (*red dots*) and npB (*green dots*), matched against proteins listed in the Human Plasma Protein Atlas (https://www.proteinatlas.org/humanproteome/blood; *grey dots*). (F) Venn diagram represents overlapping of potential protein biomarkers identified across npA and npB datasets.

### 3.4 ANK1, CAND1 and GMPR2 as malignant protein markers in ovarian and peripheral samples

Across both ovarian and peripheral sample types, ankyrin‐1 (ANK1), cullin‐associated and neddylation‐dissociated protein 1 (CAND1), and guanosine monophosphate reductase 2 (GMPR2) were consistently identified as candidate protein biomarkers (Fig. 5A; Tables S3, S4). ANK1 and CAND1 were detected as candidates in the npA dataset for both ovarian and peripheral samples, while GMPR2 was identified in ovarian serum using npB and in peripheral serum using npA (Fig. 5B). Based on these dataset‐specific findings, all three proteins were observed at lower abundances in peripheral serum, potentially due to systemic dilution (Fig. 5C). Interestingly, ANK1, CAND1 and GMPR2 were all downregulated in malignant cases across the respective sample types (Fig. 5C). Receiver operating characteristic curve analysis demonstrated good discriminatory performance between malignant and nonmalignant cases for all three proteins in ovarian samples with AUC value > 0.8 (Fig. 5D). In peripheral samples, ANK1 showed the strongest performance (AUC = 0.839), while CAND1 (AUC = 0.762) and GMPR2 (AUC = 0.778) exhibited moderate discriminatory ability (Fig. 5D). All three candidate biomarkers achieved accuracies >0.80 in both ovarian vein and peripheral serum using the corresponding ROC‐derived thresholds (Table S5), although their sensitivity and specificity profiles differed between the two sample types. ANK1 showed higher sensitivity in ovarian vein serum (83.3% vs 62.5%), whereas specificity was higher in peripheral serum (88.9% vs 95.2%) (Table S5). CAND1 and GMPR2 also showed higher sensitivity in ovarian vein serum (66.7% and 83.3%, respectively) than in peripheral serum (37.5% and 62.5%, respectively) (Table S5). Collectively, these findings support the potential of ANK1, CAND1 and GMPR2 as candidate biomarkers for distinguishing malignant from nonmalignant cases, while highlighting differences in their discriminatory characteristics between tumour‐proximal and peripheral serum.

**Fig. 5:**
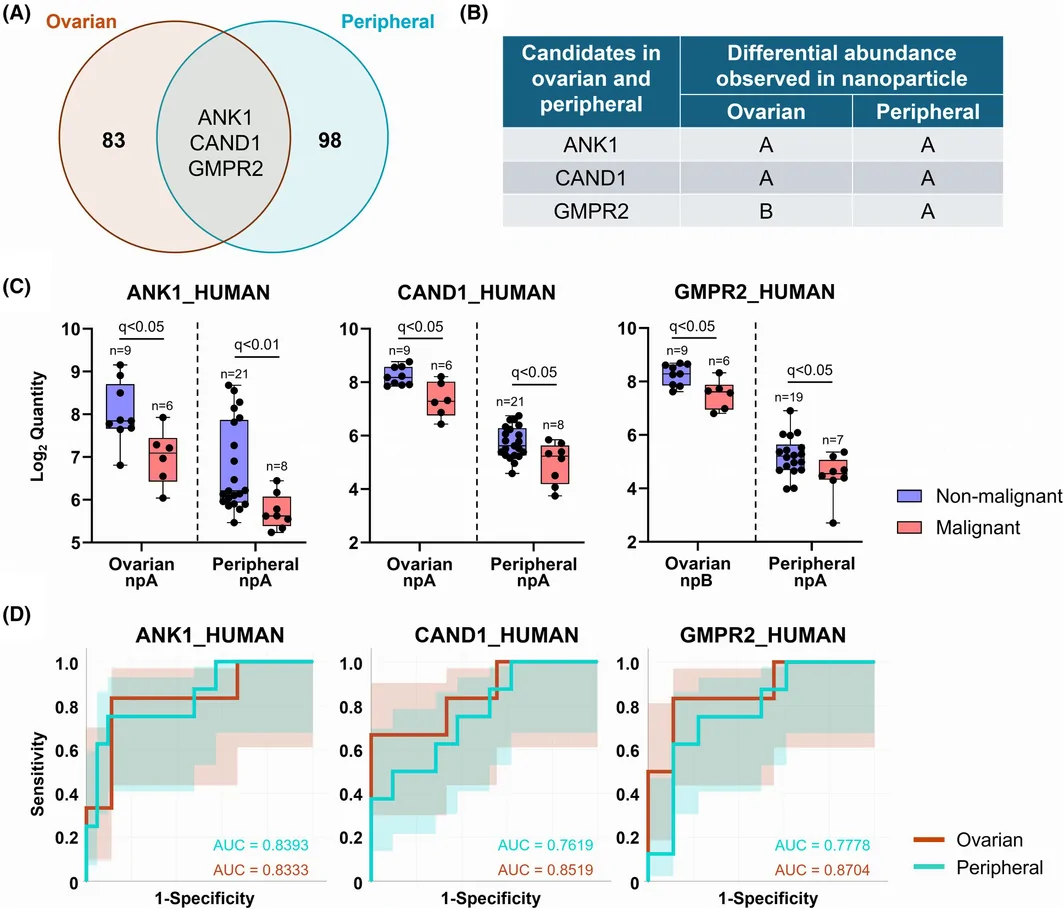
Potential biomarkers identified in both ovarian vein and median cubital vein peripheral serum. (A) Venn diagram illustrating the overlap of potential biomarkers in both sample types. (B) Table indicating the nanoparticle dataset in which differential abundance was observed for the shared candidates. (C) Boxplots comparing the log_2_‐transformed quantities of ANK1, CAND1 and GMPR2 between nonmalignant and malignant ovarian serum samples, and between nonmalignant vs malignant peripheral serum samples. Significance was assessed using false discovery rate (FDR)‐controlled unpaired Student's *t*‐tests. *q* < 0.05 and *q* < 0.01 denote an FDR of 5% and 1%, respectively. (D) Descriptive receiver operating characteristic (ROC) curve analysis of ANK1, CAND1 and GMPR2 for discrimination between malignant and nonmalignant cases in ovarian and peripheral serum.

### 3.5 Predictive performance for malignant cases in combination with clinical CA125 data

Although initial ROC curves analysis indicated that ANK1, CAND1 and GMPR2 exhibited good discriminatory potential, logistic regression was subsequently used to evaluate the discriminatory performance of these selected candidate biomarkers, individually and in combination with clinical CA125 data, using peripheral vein serum samples. Due to the absence of an independent patient cohort, repeated cross‐validation combined with permutation testing was employed to provide an internal assessment of predictive performance. Three missing values for GMPR were imputed using the feature‐wise k‐nearest neighbours (KNN) algorithm across all samples instead of within each clinical group to avoid introducing group‐specific biases during data processing. Among single markers, clinical CA125 measurements demonstrated strong classification performance (AUC = 0.927, accuracy = 0.849, *P* = 0.003), outperforming ANK1 and CAND1, while GMPR2 showed limited predictive ability (AUC = 0.618, *P* = 0.279). Combining biomarkers improved predictive performance, with CA125 + ANK1 achieving the highest discriminative ability (AUC = 0.97, accuracy = 0.91, *P* = 0.004). The improvement in AUC and predictive accuracy observed with ANK1 as compared to CA125 data alone suggest that it provides additional discriminatory information to CA125. In contrast, the inclusion of CAND1 and GMPR2 did not consistently enhance performance (Table 1), which may be attributed to overlapping biological functions, limited incremental predictive value or increased model complexity relative to the sample size. However, the wide confidence intervals across models (Table 1) indicate that these AUC estimates should be interpreted with caution given the limited sample size.

**Table 1:** Predictive performance of peripheral vein serum proteins (ANK1, CAND1 and GMPR2) for malignant cases vs nonmalignant cases in combination with clinical CA125 measurements. Performance metrics include area under the curve (AUC), 95% confidence interval (CI) and predictive accuracy estimated using repeated cross‐validation (100 repeats), with statistical significance assessed by permutation testing (*n* = 1000) using logistic regression. Analyses were performed on *n* = 26 samples (19 nonmalignant and seven malignant cases) with CA125 measurement data.

Markers	AUC	95% CI	Predictive accuracy	Permutation *P*‐value
CA125	0.927	0.804–1	0.849	0.003
ANK1	0.779	0.391–1	0.657	0.053
CAND1	0.775	0.481–1	0.704	0.053
GMPR2	0.618	0.107–0.912	0.609	0.279
CA125 + ANK1	0.97	0.786–1	0.91	0.004
CA125 + CAND1	0.877	0.536–1	0.791	0.009
CA125 + GMPR2	0.89	0.408–1	0.834	0.007
CA125 + ANK1 + CAND1	0.934	0.508–1	0.848	0.002
CA125 + ANK1 + GMPR2	0.941	0.651–1	0.872	0.006
CA125 + CAND1 + GMPR2	0.79	0.338–1	0.787	0.053
CA125 + ANK1 + CAND1 + GMPR2	0.877	0.374–1	0.827	0.012

## 4 Discussion

The limited efficacy of current biomarkers, such as CA125, for early ovarian cancer screening has called for the need to identify new and more accurate biomarkers. In this study, we aimed to identify novel candidate biomarkers for ovarian cancer malignancy using serum samples obtained from both ovarian and median cubital veins. Ovarian serum is expected to be enriched for tumour‐proximal signals, while peripheral serum may represent systemic host response to disease rather than direct tumour shedding. Therefore, a comparative analysis of malignancy‐associated candidates across these two sampling sites may facilitate the identification of biomarkers that more accurately capture tumour‐derived signals while retaining translational relevance.

While serum often presents as an attractive sample type for cancer screening, it has an extremely high dynamic range with the 22 most abundant proteins constituting 99% of total protein content, thus masking the detection of low‐abundance proteins. To overcome this limitation, the Seer Proteograph XT assay was employed to enhance proteome depth by nanoparticle‐based protein corona formation. This approach selectively enriches subsets of proteins based on their physicochemical properties, thus compressing the dynamic range and enabling improved detection of low‐abundant peptides that would otherwise remain undetected using conventional workflows. In this study, the number of identified protein groups (>5000 across all samples) substantially exceeded that reported in studies utilizing protein corona formation [31, 41] and demonstrated the increased analytical depth over neat serum [42] and other commonly used serum depletion methods, including Top14 depletion and perchloric acid precipitation [22, 43, 44]. Almost twofold more proteins were identified in ovarian vein serum as compared to the peripheral vein serum, likely reflecting the enrichment of proteins secreted locally by the ovary and/or the cancer. We observed only a limited overlap between ovarian vein and median cubital vein proteomes, indicating there were protein group differences that allowed the discernment of these two sampling sites. Proteins unique to ovarian vein serum are associated with mRNA processing, aerobic respiration and exocytosis, suggesting the presence of circulating cells released from the ovarian tumour tissue [45] while peripheral serum proteome appeared more reflective of systemic processes associated with cell survival signalling and protein quality control.

Several analytical considerations were noted in this study. First, as the assay utilizes two physicochemically distinct nanoparticle types, our results demonstrate that each nanoparticle type yields a distinct protein profile. As revealed in our downstream analysis, there was minimal overlap between candidate biomarkers identified in the npA and npB datasets. This observation suggests that each nanoparticle dataset should be treated as an independent sample rather than as complementary fractions of a single proteome. Nevertheless, the consistent direction of differential abundance between nonmalignant and malignant cases for a small subset of candidate biomarkers identified in both datasets suggests that their association with disease is robust rather than driven by nanoparticle‐specific enrichment biases. Future studies may explore the possibility of combining npA‐ and npB‐enriched peptides prior to mass spectrometric analysis to reduce downstream analytical complexity associated with managing separate datasets. In addition, recent studies have reported the undue influence of red blood cells in serum samples, which can dramatically increase sample complexity and numbers of identified proteins [17, 37, 46]. Strikingly, nanoparticle enrichment strategies are prone to platelet and peripheral blood mononuclear contamination [46]. Therefore, prior to differential analysis, we assessed the extent of potential red blood cell contamination within the serum samples of this study. Using the Baize software [37], a total of 16 experimental runs corresponding to nine samples were affected by the presence of red blood cells. These findings underscore the importance of integrating quality control measures into proteomic workflows, particularly for nanoparticle‐based serum protein enrichment strategies, to ensure data reliability and more accurate biological interpretability.

After excluding blood‐contaminated samples, we further interrogated the proteome variations between nonmalignant and malignant cases, focusing first on ovarian vein serum. Due to its close proximity to the ovarian tumour site, ovarian vein serum is likely to reflect the local tumour‐associated biological environment. This is supported by our findings that MUC16 and WFDC2, which are associated with ovarian cancer biomarkers CA125 and HE4, respectively, could only be identified in ovarian serum samples. We did not identify significant differences in MUC16 protein abundance between nonmalignant and malignant cases, despite an apparent higher abundance in malignant cases using the npB dataset. Positive associations between clinical CA125 measurements and MUC16‐derived peptides spanning multiple tandem‐repeat regions were observed in the npB dataset, suggesting that the npB measurements may better capture biologically relevant variation in MUC16 abundance. However, it is important to note that clinical CA125 was measured in peripheral serum using a chemiluminescence immunoassay, whereas MUC16 was quantified by mass spectrometry‐based proteomics in matched ovarian vein serum. As these measurements were obtained from different serum compartments using distinct analytical approaches and measure related but not directly equivalent analytes, the observed correlations should therefore be interpreted cautiously. Nevertheless, these findings provide supportive evidence that nanoparticle‐based proteomics can capture biologically relevant signals from tumour‐proximal blood, while also highlighting the importance of orthogonal validation using an independent analytical approach to establish the robustness and clinical relevance of proteomic biomarker candidates.

In ovarian serum samples, chromatin organization, megakaryocyte differentiation and nucleosome assembly were upregulated in malignant cases. Interestingly, these processes were previously found to be upregulated in native spheroids found in ascites of ovarian cancer patients, as compared to spheroids generated from ovarian cancer cell lines or adherent ascites cells, further supporting key tumour biology characteristics of ovarian cancer [47]. Specifically, upregulated levels of histones in malignant cases are consistent with reporting of extracellular histones as damage‐associated molecular pattern molecules in inflammatory diseases or cancer [48, 49, 50]. Other top upregulated biomarkers in malignant cases, including interstitial collagenase MMP1, XPO6 and GALNT2, had also been reported for their implications in the progression of ovarian and other cancer types [51, 52, 53]. Furthermore, our results suggested strong evidence for coiled‐coil domain containing protein family as biomarkers for malignant cancer, with previous studies also highlighting correlations between CCDC80 and inflammatory markers for chronic complications [54]. In peripheral serum, most candidates were found downregulated in malignant cases and were significantly associated with protein degradation and apoptosis regulation. This pattern may reflect evasion from apoptosis in malignant disease and normal tissue homeostasis for protein recycling and cell turnover in nonmalignant conditions [55]. In contrast, most upregulated candidates in malignant peripheral samples, including CCL18 chemokine and B‐type lamins, were all previously reported as potential prognostic biomarkers for ovarian cancer [56, 57].

While ovarian and peripheral serum proteomes varied significantly, with largely distinct biomarkers identified for each sample type, we observed three proteins, ANK1, CAND1 and GMPR2, that were consistently downregulated in malignant cases across both sampling sites. Similar expression trends for these proteins were observed in various cancer types. According to the Clinical Proteomic Tumour Analysis Consortium (CPTAC), ANK1 is downregulated in ovarian, kidney, colon and lung cancers as compared to normal samples [58]. In mouse osteosarcoma tumours, the overexpression of ANK1 led to suppression of cancer progression [59]. The potential of ANK1 as a blood biomarker has also been implicated in Parkinson's disease [60]. In lung cancer cells, CAND1 knockout resulted in increased cell proliferation and migration, suggesting a tumour suppressive role [61]. Loss of GMPR2 protein expression was also found to correlate with poorer survival in basal‐like tumours of breast cancer [62]. Collectively, these observations corroborate our findings and support the potential relevance of ANK1, CAND1 and GMPR2 as tumour biology‐relevant malignancy‐associated biomarkers. Among these, ANK1 demonstrated the highest discriminatory ROC score between nonmalignant and malignant cases. Importantly, classification performance improved when ANK1 was combined with clinical CA125 data, with this combination achieving the highest predictive performance across all tested combinations. Cross‐validated logistic regression combined with permutation testing further supported the robustness of this signal, highlighting the potential utility of this biomarker combination for future validation studies. Nevertheless, due to the small sample size, the lack of an independent validation dataset, the exploratory nature of the analysis, and the absence of orthogonal validation of the identified biomarker candidates, these discriminatory performances should be considered optimistic and require further validation in larger, independent cohorts. Clinical metadata, including sampling time, age, BMI and other patient‐specific characteristics, which are known to influence circulating protein levels [63, 64, 65], should also be considered to investigate potential confounding factors.

## 5 Conclusion

Ultimately, deep serum proteomics using Proteograph XT assay enabled the identification of potential low‐abundance biomarkers. Our findings highlight that ovarian vein serum enriched tumour‐proximal circulating proteins that are diluted below detectability in peripheral circulation. Hence, it is important to note that candidates that were observed in ovarian samples but not peripheral samples should not be disregarded, as a more sensitive assay such as ELISA may pick up significant differences. The minimal overlapping of protein identifications using Proteograph and other affinity‐based assays, such as the SomaScan 11 k and Olink Explore HT, may also provide complementarity in the analysis of these proteins. As the current study comprises predominantly a Stage 1 ovarian tumour cohort, future work could be expanded to include patients with both localized and metastatic disease in a longitudinal design. This would enable more robust identification of prognostic biomarkers by linking baseline molecular profiles to disease progression and clinical outcomes over time. Collectively, these findings reveal distinct proteomic signatures between tissue‐proximal and peripheral serum, advancing our understanding of how sampling location influences biomarker discovery and supporting the future development of blood‐based biomarkers for ovarian cancer diagnosis.

## Conflict of interest

The authors declare that there are no known competing financial interests or personal relationships that could have appeared to influence the work reported in this paper.

## Author contributions

MKO, DAB and PH contributed to the conceptualisation. DL, DP, CY, NC, PM and LD contributed to the methodology. DL, DP, CY and NC contributed to the data curation. DL, DP, CY, NC and PM contributed to the formal analysis. DL, DP, CY, NC, NAL and LD contributed to the investigation. DL, DP and CY contributed to the visualization. DL, DP and CY contributed to the writing—original draft. DL, CY, NAL, LD, PM, MKO, DAB and PH contributed to the writing—review and editing. NAL, MKO, DAB and PH contributed to the resources. CY, LD, DAB and PH contributed to the supervision. MKO, DAB and PH contributed to the funding acquisition. MKO, DAB and PH contributed to the project administration. All authors read and approved the final manuscript.

## Supporting information


**Fig. S1.** Comparisons of all proteins identified in this study and predefined protein lists of other affinity‐based assays.
**Fig. S2.** Comparison of protein identification and abundance correlation between npA and npB assays.
**Fig. S3.** Presence of red blood cells contamination in serum samples.
**Fig. S4.** Associations between clinical CA125 measurements from peripheral serum and ovarian serum MUC16 peptide quantities derived from proteomics.
**Fig. S5.** Top 10 malignancy‐associated biomarkers identified in ovarian vein serum.
**Fig. S6.** Candidate biomarkers identified in npA and npB datasets of ovarian serum.
**Fig. S7.** Top 10 malignancy‐associated biomarkers identified in peripheral serum.
**Fig. S8.** Candidate biomarkers identified in npA and npB datasets of peripheral serum.
**Table S1.** Clinicopathological details of patients from which serum samples were acquired for this study.
**Table S2.** Peptides associated with tandem‐repeat regions of MUC16.

**Table S3.** Proteomics analysis of ovarian vein serum samples.

**Table S4.** Proteomics analysis of peripheral vein serum samples.
**Table S5.** Receiver operating characteristic analysis of candidate biomarkers in ovarian vein and peripheral serum.

## Data Availability

Data generated in this study are available upon request from the corresponding author.
