# Supplementary material for: Tumour‐proximal deep serum proteomics identifies biomarkers associated with ovarian cancer
Source: Mol Oncol. 2026 Oct 17:10.1002/1878-0261.70351. Online ahead of print. doi: 10.1002/1878-0261.70351 (PMC13675746; doi:10.1002/1878-0261.70351)
Supplement: Supplementary file 1 [file MOL2-9999-0-s003.docx]

**SUPPLEMENTARY MATERIALS**

**Tumour-proximal deep serum proteomics identifies biomarkers associated with ovarian cancer**

Darren Lau^a^, Daniel Pincher^a^, Clifford Young^a^, Nicholas Chappell^a^, Noor A Lokman^b,d^, Parul Mittal^a^, Leigh Donnellan^a^, Martin K Oehler^b,c,d^, Doug A Brooks^a,e^, Peter Hoffmann^a,d,^*

^a^School of Pharmacy and Biomedical Science, College of Health, Adelaide University, Adelaide, SA 5000, Australia.

^b^School of Medicine, College of Health, Adelaide University, Adelaide, SA 5000, Australia

^c^Department of Gynaecological Oncology, Royal Adelaide Hospital, Adelaide, SA 5005, Australia.

^d^Robinson Research Institute, College of Health, Adelaide University, Adelaide, SA 5000, Australia.

^e^Discipline of Histopathology, School of Medicine, Trinity Translational Medicine Institute, Trinity College Dublin, Dublin, Ireland.

*Correspondence: [peter.hoffmann@adelaide.edu.au](mailto:peter.hoffmann@adelaide.edu.au)

**Supplementary Table S1. Clinicopathological details of patients from which serum samples were acquired for this study.** Age, status (H: healthy; B: benign; M: malignant), diagnosis, stage and grade (where applicable), site of serum collection (ovary or peripheral) and approved ethics associated with each patient ID.

| **Patient ID** | **Age** | **Status** | **Diagnosis** | **Sampling**  **date** | **Stage** | **Grade** | **Ovary**  **(Passed QC- Y/N)** | **Peripheral**  **(Passed QC – Y/N)** | **Associated RAH protocol number** | **Clinical**  **peripheral CA125 measurements** |
| --- | --- | --- | --- | --- | --- | --- | --- | --- | --- | --- |
| 1624 | 48 | H | Normal control | 12/12/2014 |  |  | ✓ (Y) | ✓ (Y) | 140101 | 9 |
| 1836 | 60 | H | Normal control | 20/10/2015 |  |  |  | ✓ (Y) | 140101 | 3 |
| 1839 | 44 | H | Normal control | 20/10/2015 |  |  |  | ✓ (Y) | 140101 | 7 |
| 1840 | 65 | H | Normal control | 20/10/2015 |  |  |  | ✓ (Y) | 140101 | 5 |
| 1844 | 48 | H | Normal control | 23/10/2015 |  |  |  | ✓ (Y) | 140101 | 27 |
| 1845 | 54 | H | Normal control | 23/10/2015 |  |  |  | ✓ (Y) | 140101 | 5 |
| 1963 | 30 | H | Normal control | 07/06/2016 |  |  |  | ✓ (Y) | 140101 | 7 |
| 1972 | 48 | H | Normal control | 24/06/2016 |  |  |  | ✓ (Y) | 140101 | 20 |
| 1993 | 37 | H | Normal control | 13/09/2016 |  |  |  | ✓ (Y) | 140101 | 25 |
| 2311 | 56 | H | Normal control | 16/10/2020 |  |  |  | ✓ (Y) | HREC/18/  CALHN/811 | 5 |
| 986 | 48 | B | Benign left ovarian cyst | 21/09/2012 |  |  | ✓ (Y) | ✓ (Y) | 080102 | 77 |
| 1193 | 67 | B | Benign mucinous cystadenoma of the ovary | 14/05/2013 |  |  | ✓ (N) | ✓ (Y) | 080102 | 7 |
| 1324 | 63 | B | Benign serous cystadenoma of the ovary | 24/09/2013 |  |  | ✓ (Y) | ✓ (Y) | 080102 |  |
| 1519 | 35 | B | Benign ovarian cysts | 30/05/2014 |  |  | ✓ (Y) | ✓ (Y) | 140101 | 10 |
| 1539 | 86 | B | Bilateral benign ovarian fibroadenomas | 16/06/2014 |  |  | ✓ (Y) | ✓ (Y) | 140101 | 98 |
| 1546 | 75 | B | Benign serous cystadenoma of the ovary | 30/06/2014 |  |  | ✓ (Y) | ✓ (N) | 140101 | 20 |
| 1592 | 42 | B | Benign mucinous cystadenoma of the ovary | 21/10/2014 |  |  | ✓ (N) | ✓ (Y) | 140101 |  |
| 1617 | 54 | B | Benign dermoid and benign mucinous cystadenoma of the ovary | 02/12/2014 |  |  | ✓ (Y) | ✓ (Y) | 140101 | 12 |
| 1633 | 76 | B | Benign serous cystadenomas of both ovaries | 20/01/2015 |  |  | ✓ (Y) | ✓ (Y) | 140101 | 9 |
| 1676 | 66 | B | Benign serous cystadenoma of the ovary | 31/03/2015 |  |  | ✓ (N) | ✓ (Y) | 140101 | 5 |
| 1723 | 75 | B | Benign serous cystadenofibroma of the ovary | 11/05/2015 |  |  |  | ✓ (Y) | 140101 | 17 |
| 1732 | 54 | B | Benign serous cystadenofibroma of the ovary | 19/05/2015 |  |  | ✓ (Y) | ✓ (Y) | 140101 | 10 |
| 1955 | 63 | B | Benign mucinous cystadenoma of the ovary | 23/05/2016 |  |  | ✓ (N) |  | 140101 | 319 |
| 916 | 71 | M | Endometrioid adenocarcinoma of the ovary | 17/07/2012 | 1a | 1 | ✓ (Y) | ✓ (Y) | 080102 | 144 |
| 946 | 44 | M | Endometrioid adenocarcinoma of the ovary | 13/08/2012 | 1a | 2 | ✓ (N) | ✓ (Y) | 080102 | 81 |
| 1146 | 25 | M | Endometrioid adenocarcinoma of the ovary | 25/03/2013 | 1a | 2 | ✓ (Y) | ✓ (N) | 080102 | 111 |
| 1288 | 65 | M | Serous carcinoma of the ovary | 13/08/2013 | 3 | 3b | ✓ (N) | ✓ (Y) | 080102 | 25 |
| 1368 | 72 | M | Endometrioid adenocarcinoma of the ovary | 29/12/2013 | 1a |  | ✓ (N) | ✓ (Y) | 080102 |  |
| 1575 | 46 | M | Serous papillary carcinoma of the ovary | 05/09/2014 | 1c | 3 | ✓ (Y) | ✓ (Y) | 140101 | 39 |
| 1665 | 38 | M | Serous papillary carcinoma of the ovary | 10/03/2015 | 1a | 3 | ✓ (Y) | ✓ (Y) | 140101 | 190 |
| 1873 | 78 | M | Bilateral serous carcinomas of the ovary and serous carcinoma of the fallopian tube | 30/11/2015 | 2a | 3 | ✓ (Y) | ✓ (Y) | 140101 | 440 |
| 2008 | 64 | M | Clear cell carcinoma of the ovary | 24/10/2016 | 1c | 3 | ✓ (Y) | ✓ (Y) | 140101 | 74 |

**Supplementary Table S2. Peptides associated with tandem repeat regions of MUC16.** Abundances of peptides that correspond to tandem repeat regions of MUC16 from ovarian serum samples with matched clinical CA125 measurements from peripheral serum. PDPTGPGLDR is only present in one tandem repeat region, while the others are present in at least five.

| **Patient ID** | **CA125** | **PDPTGPGLDR** | **LTLLRPEK** | **NTSVGPLYSGCR** | **DGAATGVDAICTHR** | **ELGPYTLDR** |
| --- | --- | --- | --- | --- | --- | --- |
|  |  | ***npA*** | | | | |
| 1519 | 10 | 2842.212158 |  | 15.5870533 |  |  |
| 1539 | 98 | 2087.590332 |  |  |  |  |
| 1617 | 12 | 1858.267456 |  |  |  |  |
| 1624 | 9 | 2890.959717 |  |  |  |  |
| 1633 | 9 | 2661.681396 |  |  | 10.24121761 |  |
| 1732 | 10 | 2216.154541 |  |  |  |  |
| 986 | 77 | 1507.195679 |  |  |  |  |
| 1575 | 39 | 912.9585571 |  |  |  |  |
| 1665 | 190 | 2532.016357 |  |  |  |  |
| 1873 | 440 | 2589.491455 |  |  | 21.33661652 |  |
| 2008 | 74 | 1396.154175 |  |  |  |  |
| 916 | 144 | 1308.575928 |  |  |  |  |
|  |  |  |  |  |  |  |
|  |  | ***npB*** | | | | |
| 1519 | 10 | 77.79323578 | 29.15394783 | 45.27571106 | 9.729863167 | 33.26997375 |
| 1539 | 98 |  |  |  |  |  |
| 1617 | 12 | 111.9692841 | 98.67275238 | 125.6315918 | 32.89044666 | 141.6496735 |
| 1624 | 9 | 318.1159668 | 11.5321188 | 67.97528839 | 6.186566353 | 48.90418243 |
| 1633 | 9 | 150.3087463 | 22.0278511 | 50.45078659 | 19.34131813 | 49.90914154 |
| 1732 | 10 |  |  |  |  |  |
| 986 | 77 | 68.84048462 | 26.35985184 |  |  |  |
| 1575 | 39 |  |  |  |  |  |
| 1665 | 190 | 69.98867035 | 7.721848965 | 131.9163818 | 37.22232342 | 97.90858459 |
| 1873 | 440 | 113.819519 | 43.31495285 | 145.1136322 | 38.46808577 | 111.728714 |
| 2008 | 74 |  | 54.87541199 | 48.84850311 | 19.35682297 | 44.26236343 |
| 916 | 144 | 86.29673004 | 43.49765396 |  |  |  |

**Supplementary Table S3. Proteomics analysis of ovarian vein serum samples.** Abundances (in log2) of protein groups identified in ovarian vein serum samples without red blood cell contamination using npA and npB and whether they were identified as malignancy-associated candidate biomarkers.

*Supplementary Table S3 is presented in a separate Excel file.*

**Supplementary Table S4. Proteomics analysis of peripheral vein serum samples.** Abundances (in log2) of protein groups identified in peripheral vein serum samples without red blood cell contamination using npA and npB, and whether they were identified as malignancy-associated candidate biomarkers.

*Supplementary Table S4 is presented in a separate Excel file.*

**Supplementary Table S5. Receiver operating characteristic analysis of candidate biomarkers in ovarian vein and peripheral serum.** Sensitivity, specificity, positive predictive value (PPV), negative predictive value (NPV), and accuracy are provided (based on **Fig. 5D**).

|  | **ANK1** | | **CAND1** | | **GMPR2** | |
| --- | --- | --- | --- | --- | --- | --- |
|  | **Ovarian** | **Peripheral** | **Ovarian** | **Peripheral** | **Ovarian** | **Peripheral** |
| AUC | 0.8333 | 0.8393 | 0.8519 | 0.7619 | 0.8704 | 0.7778 |
| Threshold (log_2_) | 7.2115 | 4.0726 | 7.2462 | 4.0726 | 7.6459 | 4.4692 |
| Sensitivity | 0.8333 | 0.625 | 0.6667 | 0.375 | 0.8333 | 0.625 |
| Specificity | 0.8889 | 0.9524 | 1 | 1 | 0.8889 | 0.8889 |
| PPV (Precision) | 0.8333 | 0.8333 | 1 | 1 | 0.8333 | 0.7143 |
| NPV | 0.8889 | 0.8696 | 0.8182 | 0.8077 | 0.8889 | 0.8421 |
| Accuracy | 0.8667 | 0.8621 | 0.8667 | 0.8276 | 0.8667 | 0.8077 |

**Supplementary Fig. S1. Comparisons of all proteins identified in this study and predefined protein lists of other affinity-based assays.** Venn diagram showing the overlapping of protein group identifications (based on UniProtIDs) from this study, and the content of Olink Explore HT and SomaScan 11k assays available online.

**Supplementary Fig. S2. Comparison of protein identification and abundance correlation between npA and npB assays.** (A) Number of protein groups identified in individual ovarian serum sample using npA (*purple*) and npB (*green*). (B) Number of protein groups identified in individual peripheral serum sample using npA (*purple*) and npB (*green*). (C) Overlap of protein groups identified by npA and npB in ovarian serum samples. (D) Overlap of protein groups identified by npA and npB in peripheral serum samples. (E) Spearman correlation of protein abundance measurements for commonly identified proteins (3,779 proteins) between npA and npB in ovarian serum samples. (F) Spearman correlation of protein abundance measurements for commonly identified proteins (1,767 proteins) between npA and npB in peripheral serum samples.

**Supplementary Fig. S3. Presence of red blood cells contamination in serum samples.** Visual observations of (A) ovarian and (B) peripheral samples with the indication of blood contamination reported by Baize software highlighted in red.

**Supplementary Fig. S4. Associations between clinical CA125 measurements from peripheral serum and ovarian serum MUC16 peptide quantities derived from proteomics.** Correlation plots for the quantities of peptides with sequences LTLLRPEK, NTSVGPLYSGCR, DGAATGVDAICTHR and ELGPYTLDR that are found in at least 5 MUC16 tandem repeats and clinical measurements of peripheral CA125 in log_10_. r, Spearman correlation.

**Supplementary Fig. S5. Top 10 malignancy-associated biomarkers identified in ovarian vein serum.** Whisker box plots showing the log₂-transformed abundances of the top 10 malignancy-associated protein candidates, ranked by fold change, comparing non-malignant and malignant cases in the (A) npA and (B) npB datasets. Significance was assessed using FDR-controlled unpaired Student’s t-tests. Statistical significance is reported as *q*-values. q<0.05 and q<0.01 denote an FDR of 5% and 1%, respectively.

**Supplementary Fig. S6. Candidate biomarkers identified in npA and npB datasets of ovarian serum.** Whisker box plots showing the log₂-transformed abundances of the protein candidates comparing non-malignant and malignant cases in npA and npB datasets. Significance was assessed using FDR-controlled unpaired Student’s t-tests. Statistical significance is reported as *q*-values. q<0.05 and q<0.01 denote an FDR of 5% and 1%, respectively.

**Supplementary Fig. S7. Top 10 malignancy-associated biomarkers identified in peripheral serum.** Whisker box plots showing the log₂-transformed abundances of the top 10 malignancy-associated protein candidates, ranked by fold change, comparing non-malignant and malignant cases in the (A) npA and (B) npB datasets. Significance was assessed using FDR-controlled unpaired Student’s t-tests. q<0.05 and q<0.01 denote an FDR of 5% and 1%, respectively.

**Supplementary Fig. S8. Candidate biomarkers identified in npA and npB datasets of peripheral serum.** Whisker box plots showing the log₂-transformed abundances of the protein candidates comparing non-malignant and malignant cases in npA and npB datasets. Significance was assessed using FDR-controlled unpaired Student’s t-tests. q<0.05 and q<0.01 denote an FDR of 5% and 1%, respectively.
